# Analysis of HIC and Hydrostatic Pressure in the Human Head during NOCSAE Tests of American Football Helmets

**DOI:** 10.3390/brainsci11030287

**Published:** 2021-02-25

**Authors:** Mateusz Dymek, Mariusz Ptak, Monika Ratajczak, Fábio A. O. Fernandes, Artur Kwiatkowski, Johannes Wilhelm

**Affiliations:** 1 Faculty of Mechanical Engineering, Wroclaw University of Science and Technology, Lukasiewicza 7/9, 50-371 Wroclaw, Poland; 2 Faculty of Mechanical Engineering, University of Zielona Gora, ul. Szafrana 4, 65-516 Zielona Gora, Poland; m.ratajczak@iizp.uz.zgora.pl; 3 TEMA—Centre for Mechanical Technology and Automation, Department of Mechanical Engineering, Campus de Santiago, University of Aveiro, 3810-193 Aveiro, Portugal; fabiofernandes@ua.pt; 4 Department of Neurosurgery, Provincial Specialist Hospital in Legnica, ul. Iwaszkiewicza 5, 59-220 Legnica, Poland; artur.kwiatkowski@szpital.legnica.pl; 5 CFturbo GmbH, Unterer Kreuzweg 1, 01097 Dresden, Germany; johannes.wilhelm@cfturbo.com

**Keywords:** brain injury, finite element head model, American football, helmet, helmet testing, biomechanics, head injury criterion, concussion, NOCSAE

## Abstract

Brain damage is a serious economic and social burden. Contact sports such as American football, are one of the most common sources of concussions. The biomechanical response of the head–helmet system caused by dynamic loading plays a major role. The literature has focused on measuring the resultant kinematics that act on the head and helmet during tackles. However, few studies have focused on helmet validation tests, supported by recent findings and emerging numerical approaches. The future of helmet standards could benefit from insights at the level of injury mechanisms, using numerical tools to assess the helmets. Therefore, in this work, a numerical approach is employed to investigate the influence of intracranial pressure (ICP) on brain pathophysiology during and after helmeted impacts, which are common in American football. The helmeted impacts were performed at several impact locations according to the NOCSAE standard (configurations A, AP, B, C, D, F, R, UT). In order to evaluate the ICP levels, the αHEAD finite element head and brain model was combined with a Hybrid III-neck structure and then coupled with an American football helmet to simulate the NOCSAE impacts. In addition, the ICP level was analyzed together with the resulting HIC value, since the latter is commonly used, in this application and others, as the injury criterion. The obtained results indicate that ICP values exceed the common threshold of head injury criteria and do not correlate with HIC values. Thus, this work raises concern about applying the HIC to predict brain injury in American football direct head impacts, since it does not correlate with ICP predicted with the FE head model.

## 1. Introduction

American football is a prominent sport, yet it is also considered a high-risk competitive game [1]. Despite the mandatory usage of protective gear such as helmets and shoulder pads, severe injuries still frequently occur from direct impacts [2]. This issue is a vibrant topic not only across the United States and Canada, where football is one of the most popular sports, but also in some European countries since its popularity has increased significantly. The rates of traumatic brain injuries (TBI) in professional players are high, and so is the number of former players diagnosed with chronic traumatic encephalopathy (CTE) [3,4,5,6]. CTE is a severe brain disorder caused by repeated head trauma that is associated with brain degeneration. The development and evolution, and therefore diagnosis, of this condition take time. Diagnosis is usually suspected with symptoms such as memory loss, impaired judgment, aggression and depression [1,7,8,9].

The brain damage guidelines are based on the head impact criterion (HIC), which is a coefficient based on the resultant linear acceleration in the head’s center of gravity. It should be noted that this criterion does not take into account the rotational component and neglects more complicated motions of the highly complex head–neck–torso system. National Operating Committee on Standards for Athletic Equipment (NOCSAE) certified helmets must not exceed a threshold severity index (SI), which is based on linear acceleration like HIC, but also does not allow the head to exceed a threshold for rotational acceleration. Nevertheless, these criteria need to be seen as simplifications of the complex initial motion and preferably find their validation in empirical data and expressions of injury outcome without the need to describe in-depth injury mechanisms.

On the other side of the assessment, the criterion for helmet validation tests finds its physical base in a dummy representing a human body [10,11]. This includes the NOCSAE headform, which was obtained by reconstructing computed tomography scans. The model is simplified and includes only a head skin rubber layer, a rigid skull and a rigid neck. The internal structures are not represented. By using a different approach to physical head and neck structures, the Hybrid III Head-Neck (HIII) model was obtained from technical drawings published by the U.S. Department of transportation. The model consists of a head skin rubber layer, rigid skull, head mount and parts representing the neck, such as occipital condyle pin joints, neck butyl rubber discs, aluminum discs, a neck cable and a nodding block.

In general, helmets consist of a comfort liner and an energy absorption liner, covered by a stiff outer shell. The last two components are responsible for most of the impact energy dissipation and absorption [12,13]. The shells and paddings currently used in football helmets are made of polymeric materials, which are typically thermoplastic for the outer element and viscoelastic foams for the paddings.

We have noted the limited character of the assessment procedures, which rely on predominantly rigid head forms and simplified neck structures in the physical domain. We can overcome this limitation by including a numerical head and brain model that relates to injury criteria. Currently, the finite element (FE) method is often used to study neurotrauma and continues to emerge as a useful tool in the field of neuroscience [14]. However, despite different criteria, such as Head Impact Power (HIP), Brain Injury Criterion (BrIC) or the use of different finite element head models such as Global Human Body Models Consortium (GHBMC), Simulated Injury Monitor (SIMon) or Louis Pasteur University (ULP), there is still limited research in validation tests on brain behaviour during the impact [15,16,17,18,19,20].

Regarding research on the role of football helmets in injury prevention, finite element models of helmets have now found increasing use as research tools to investigate stress distribution and energy absorption [21,22,23,24,25,26,27]. There are numerous validated helmet models under a wide range of impact conditions. The energy distribution and absorbing materials are studied to improve the design process and evaluate their performance. Moreover, studies have focused on the point of impact and corresponding overload acting on the dummy head [22,28,29,30,31,32]. In the literature, there is a tendency to focus on technological aspects of helmets and the assessment of players safety [33,34,35]. Recently, Hernandez et al. [34] investigated the hypothesis of damage to the corpus callosum caused by motions of the falx cerebri due to coronal and horizontal rotations. Using FE simulations with a head model to correlate with two diagnosed sports-related concussions, a unique relationship was observed between the corpus callosum and falx cerebri, concluding that the corpus callosum may be sensitive to coronal and horizontal rotations. More studies have been performed using computational models of the human head, dummies and helmets, and test methods to assess the performance of American football helmets [19,36,37,38] or tackles in other full-contact team sports, such as rugby union [39,40,41,42]. Currently, this research field is of the highest importance, as demonstrated by the involvement of several research groups who developed open-source dummy and impactor models [43]. This is also valid for helmets, with studies focusing on material characterization and constitutive model calibration, followed by helmet validation [21,22,44].

While the current norms and regulations are based primarily on accelerations and rigid test equipment, researchers have already attempted to connect the strain in the cortex to the injury outcome [45,46]. Therefore, in the present study, the authors investigate intracranial pressure (ICP) during the linear impact of a football helmet and relate the calculated results to the original injury threshold of ICP. A comparison between the ICP-based and conventional HIC-related results shows the limitations of both approaches.

### Medical Background

For many years concussion was thought to be caused by blunt forces, with only mild, functional brain disturbance with no long-term health impact. There are no specific direct physiologic measures for detecting concussion and diagnosis is based on a wide variety of physical, cognitive, and emotional symptoms and aberrations of the circadian rhythm. Concussion is distinguished from other mild brain traumatic injuries by the absence of any structural abnormality in so-called standard medical imaging (computed tomography and standard field magnetic resonance) [47]. However, recent developments in the field of magnetic resonance imaging have provided new evidence about some profound pathological changes present in the nerve cells of individuals diagnosed with a concussion.

Even mild traumatic brain injury is now known to cause more than just a flux of sodium, potassium and calcium ions [48]. Regional alterations in cerebral blood flow and oxygen utilization observed many days after the injury suggest compromised metabolism and lack of full recovery [49]. Additionally, some studies incorporating high field 7T magnetic resonance showed blood products in brain parenchyma, which is clear evidence of structural damage at a microscopic scale [50].

One of the most famous examples of players suffering from CTE was a former New England Patriots star, Aaron Hernandez, who committed suicide in prison. His autopsy revealed that he was suffering from a severe case of CTE, already categorized as stage 3 [51,52]. This did not seem to be an isolated case, since a recent study reported 177 players diagnosed with CTE out of a total of 202 [53]. By investigating the background sport career of these 202 players, 111 were former National Football League (NFL) players and almost all of them (110 players) were diagnosed with CTE [53]. More frequently used in the discussion about contact sports and the related brain injuries is TBI, which embarrasses injuries caused by a jolt or a blow to the head from a collision and penetrating trauma or indirect head impacts (e.g., impacts to the torso) [54,55,56].

Contemporary “return to play” policy, based on the 5th edition of Sport Concussion Assessment Tool (SCAT5) and similar symptom-based approaches, reflects empirical knowledge about the duration and depth of cerebral abnormalities [57]. Some rare and deadly conditions, such as “second impact syndrome” [58], give further clues about more persistent effects than just an ion flux and warrant extra precautions following even a mild concussion, despite symptom resolution.

**Figure 1:**
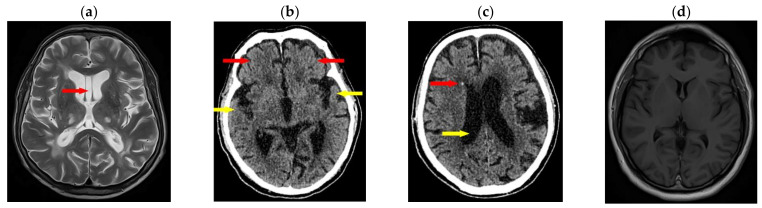
Typical findings in chronic traumatic encephalopathy: (**a**) A 58-year-old male with a history of repetitive trauma MRI in T2 sequence; cave of septum pellucidum (red arrow) is visible between enlarged lateral ventricles, (**b**) Advanced atrophy of frontal and temporal lobes (red and yellow arrows respectively) visible in plain CT scans, (**c**) Enlargement of ventricles in atrophied brain (yellow arrow); calcification (red arrow), suggestive of a neurodegenerative process, (**d**) MRI of a healthy person for reference.

Repeated impacts to the head can have cumulative effects, resulting in the well-known, but pathologically not very well explained, chronic traumatic encephalopathy. Symptoms of dementia, motor dysfunction in pyramidal and cerebellar pathways, and personality changes occur after many years of professional football, boxing, hockey and many other contact sports. The clinical picture of this encephalopathy reassembles Alzheimer’s disease to some extent. Postmortem examination of symptomatic athletes with a history of repeated concussions revealed many similar pathological findings in 92% of them, including cerebral atrophy, degeneration of various brain areas, deposition of beta-amyloid protein and tau, and cavum septum pellucidum [58,59]. Despite the fact that indisputable diagnosis of chronic traumatic encephalopathy can only be made after death, radiological evidence of non-age-related degenerative changes are often present (Figure 1).

## 2. Materials and Methods

### 2.1. Model Discretization

#### 2.1.1. αHEAD Finite Element Head Model

**Figure 2:**
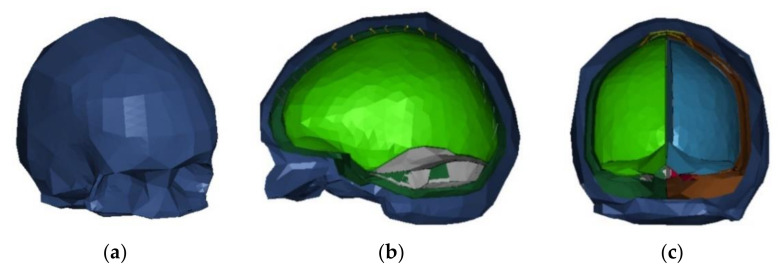
αHEAD model: (**a**) isometric view, (**b**) sagittal section view through the skull, (**c**) coronal section view through the skull.

In this study, we used a validated numerical model of the head published by Ratajczak et al. [60]; the αHEAD finite element head and brain model (Figure 2). The model includes a system of bridging veins with a distinction made between mechanical properties in different parts of the head [61]. Moreover, compared to Yet Another Head Model (YEAHM), Global Human Body Models Consortium (GHBMC) or the current Advanced Head Models for Safety Enhancement and Medical Development (αHEAD) Finite Element Head Models, αHEAD enables one to obtain valid data while saving computational time and resources [18,62,63]. Additionally, it compares HIC values obtained with the HIII head-neck model to intracranial pressure with αHEAD. The helmet model was developed and validated by Biomechanical Consulting and Research, LLC (Biocore) [43,64]. The research was conducted by using the LS-DYNA (LSTC, Livermore, CA, USA) environment. The 3D geometric model of the brain and skull was developed based on medical images acquired from medical scanners. The created 3D object was exported to the stereolithography format (STL), which enabled the authors to proceed with digital processing using computer-aided class programs such as CATIA v5 and MeshLab. The use of the ELFORM13, i.e., element formulation options in LS-DYNA, enabled the authors to eliminate the volume locking phenomenon in this model. The ELFORM13 element formulation options prevented volumetric locking in the model by defining the nodal volumes and evaluating the average nodal pressures in terms of these volumes. The brain’s geometry was divided into four parts: the right/left hemisphere of the brain and the right/left cerebellum. The model is also advantageous in terms of biofidelity, in instances such as dura mater, cerebrospinal fluid (CSF), falx cerebri and cerebellar tentorium, superior sagittal sinus and bridging veins. The bridging veins’ geometrical parameters and their distribution were developed on the basis of the descriptions in Oka [60] and Kleiven [65]. In the numerical model, the bridging veins were distinguished between the frontal, parietal and occipital parts. Mechanical properties used in this study are summarised in Table 1 and Table 2.

#### 2.1.2. Hybrid III Head-Neck Model

**Figure 3:**
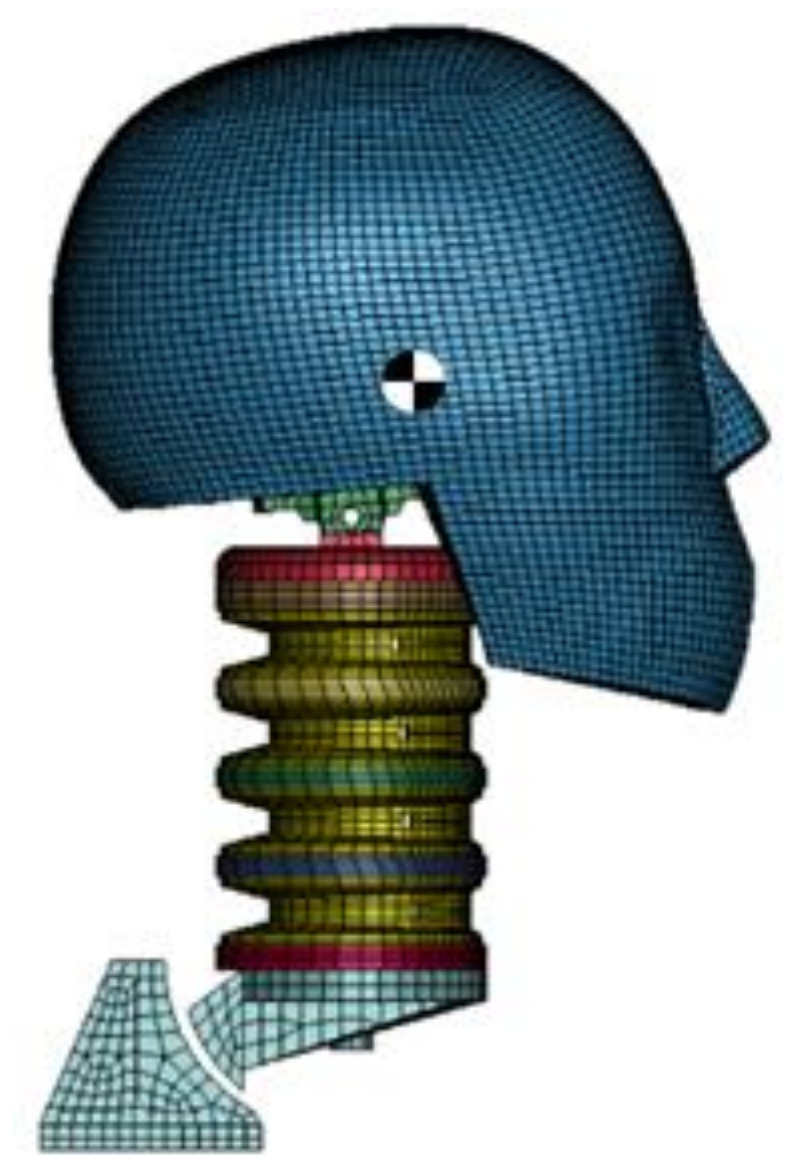
Hybrid III head-neck model with marked center of gravity.

The investigated approach simulations were performed using the Hybrid III head-neck model. As Figure 3 depicts, the model is composed of a skull, head skin, neck and neck mount. The center of gravity of the head model is marked in Figure 3. In addition, a local head accelerometer is present at this point. The accelerometer gives local head acceleration data. The overall mass is equal to 5.74 kg [64].

#### 2.1.3. Helmet and Impactor Model

**Figure 4:**
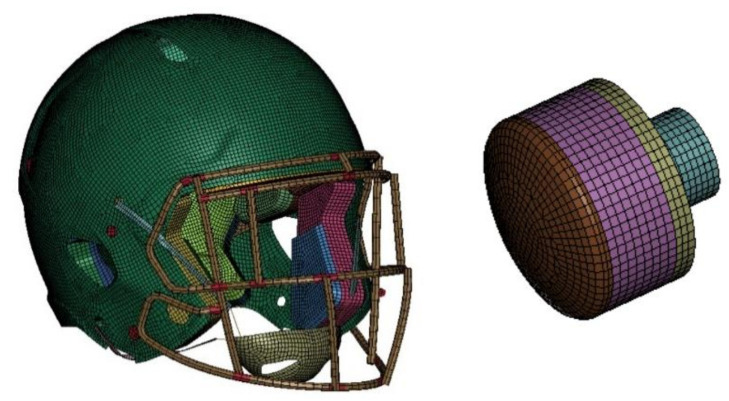
2016 Riddell Speed Classic helmet and the impactor discrete model used in the study.

The reverse engineering approach was used to make the discrete helmet model (2016 Riddell Speed Classic). Mechanical properties obtained in individual material tests are presented in Table 3. Each part was modelled individually in order to obtain the best quality mesh. The overall mass of the helmet is 1.98 kg (Figure 4). In comparison, the physical model weights 2.3 kg. The following simplifications were used to minimize computational time:

- In the real model, the padding is covered in plastic covers that can be pumped with air. In the discrete model, only a plastic sheet is modelled at the back of the pads. During component validation, the difference was found to be minimal.
- The comfort foam stiffness was increased by a factor of 2.5 compared to the experimental results to prevent instabilities. However, when evaluating the two stiffnesses for some linear impactor simulations, this difference was negligible.
- The foam material can be sensitive to weather conditions such as temperature or penetrating trauma.

The impactor was developed and validated by Biomechanical Consulting and Research, LLC (Biocore) [43,64]. The impactor model is composed of five different parts, and its overall mass is 15.4 kg [64].

### 2.2. Simulation Setup

**Figure 5:**
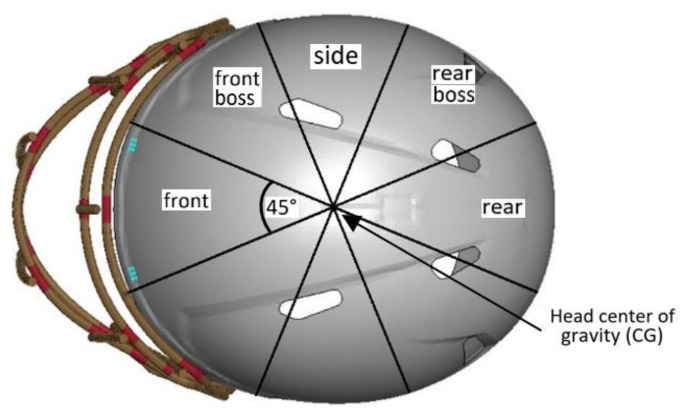
Impact location bins (based on [70]).

The idea behind the NOCSAE validation test is to ensure that all athletic equipment meets the standards, with the aim to enhance athletic safety. There are various categories for different equipment tested. One of the major problems that the organization encountered was addressing head and neck injuries in football. The concept in 1970 was to test the system with a humanoid head to investigate the degree of hazard experienced by players. The year 1975 was revolutionary for the helmet validation system because drop test equipment was installed and used by one of the largest helmet reconditioning facilities for testing helmets. Nowadays, testing has been broadened to include a pneumatic ram impactor, delivering impacts in selected locations (Figure 5). The NOCSAE organization states that helmets should be reconditioned every two years in order to minimize the probability of damage. All reconditioned and manufactured helmets must meet the latest standards; the newest was introduced in November 2019. All helmets that passed the tests are marked with a “SEI certified” sign. The manufacturers and NOCSAE organization underline that no helmet completely prevents injuries [69].

**Figure 6:**
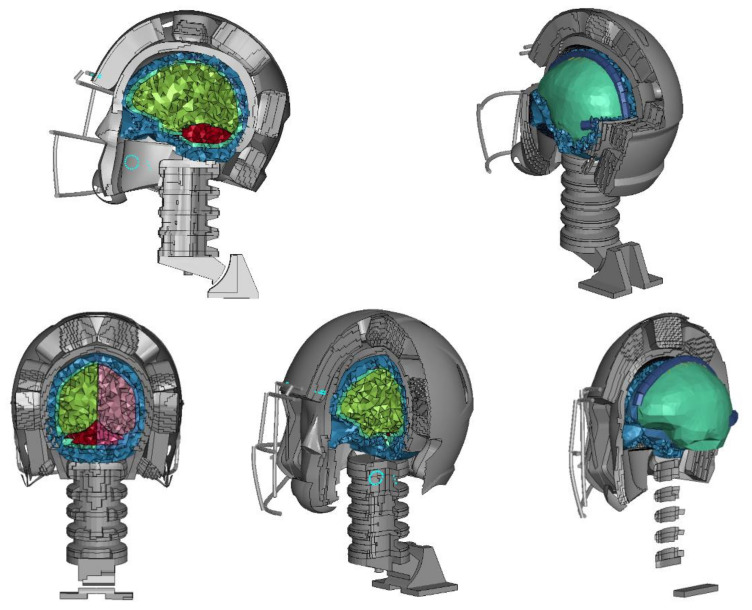
Selected views of the helmet model (grey) with the αHEAD head model (colors).

In order to analyze the behavior of the brain during the tests, it was necessary to combine a helmet model with the αHEAD model (Figure 6). The connection between the neck geometry and the skull, which is the nodding joint at the top of the neck connected to the adapter at the bottom of the head with a pin, remained the same as in the Hybrid HIII model. A change made on the skull geometry was that the neurocranium was replaced with the αHEAD model. The replicate movements at the occipital condyle were not changed. The connection between the neurocranium (αHEAD) and the facial skeleton (Hybrid HIII with the adapter) is rigid (LS-DYNA: CONSTRAINED-RIGID BODIES card). The geometry of the αHEAD skull was tied to the layer representing the skin of Hybrid HIII. The remaining contacts between the helmet and the skin layer remained unchanged. To compare the results to actual validation tests, all configurations were simulated with the Hybrid HIII head-neck model and αHEAD. Eight simulations were performed, each with a different point of contact (all according to Biocore analysis (Table 4) [68]). In each simulation, the moving part was the impactor with an initial velocity of 9.3 m/s. It was chosen because it gave the highest test speed, in order to verify the worst-case scenario in terms of the impact loading. The model is composed of a discrete helmet model, Hybrid III Head-Neck Model with implemented αHEAD model, and a neck mount that is set as rigid in all degrees of freedom.

## 3. Results

The primary goal of this study was to examine the effect of impact location on HIC (measured with respect to the head’s center of gravity) and intracranial pressure in football helmets. The impact locations are in accordance with NOCSAE standards with a fixed initial impactor velocity. Kinematic parameters such as velocities, accelerations and impact locations may correlate with brain strain, yet further exploration of this was not the intent of this study. Moreover, as helmets are meant to protect the head and brain, neck injury and normalized neck injury criteria were also not the focus of this research.

The highest HIC value was recorded in configuration AP (650) and R (731) (Table 5, which corresponds to the frontal and rear locations, respectively). As mentioned before, HIC is based on the longitudinal acceleration, which is why the registered values are of high magnitude. The lowest HIC value was recorded in configuration UT (304). The simulation showed that the head injury criterion and severity index (SI), which are both based on acceleration, are limited in their expression regarding different impact locations, just as only a low percent of direct (translational) impact occurs. HIC is calculated as an integral of resultant translational acceleration over a specified time window, and will naturally increase when a direct hit or impact occurs, as the loss of velocity within a very short period of time gains significant weight, but does not induce a significant amount of rotation. Most impacts in sports, racing or urban situations are oblique, which is why the parameter is lower, while values of rotational acceleration increase. The values of rotational acceleration acting on the head need to be pointed out. Bearing in mind the fact that skull bones are more vulnerable to side impacts than longitudinal impacts, this can have a significant effect [71].

This study proves that even though HIC values are permissible (HIC threshold value is 1000), more sophisticated parameters such as the presented intracranial pressure exceed threshold values (~237 kPa) in each configuration (Table 5) [45,69]. It is necessary to point out that αHEAD is based mainly on a tetrahedral mesh approach. Due to its structure (high-pressure values at the boundary nodes), the pressure values are averaged using LS-DYNA’s nodal averaging technique. With that being said, validation tests of helmets should be performed with much more strict and demanding rules. Another suggestion would be to use a THUMS (Total Human Model for Safety) dummy head as it was developed with a simplified brain instead of a simple magnesium alloy head model. With today’s technology, any investigation should include a higher level of parameters. In the authors’ opinion, a helmet for sport, urban or safety purposes, should be certified under more strict criteria than HIC or the severity index (SI). Our results show that it is necessary to continue analyzing the effects on the brain in a helmet in order to minimalize the probability of injury. Additionally, using the newest absorbing technology, it will be possible to design new, more protective helmets.

Moreover, the helmet shell and facemask remained intact in all configurations; no damage to the skull structure was reported. Based on these additional criteria, one can presume that athletes’ body parts such as eyes, nose, mouth or ears are completely safe.

It is necessary to point out that the αHEAD discrete model does not include the brain’s full vascular structure, which can influence tissue behavior [60]. Nevertheless, this model was sufficient to prove initial assumptions about intracranial pressure exceeding threshold values, while maintaining HIC at permissible values.

### Configuration A

**Figure 7:**
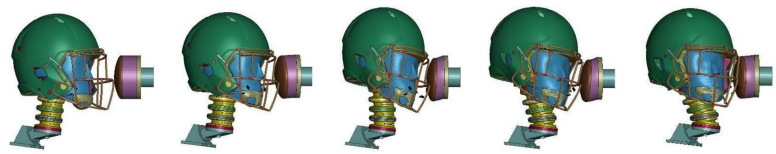
Course of simulation (0–20 ms, 5 ms interval) in configuration A.

**Figure 8:**
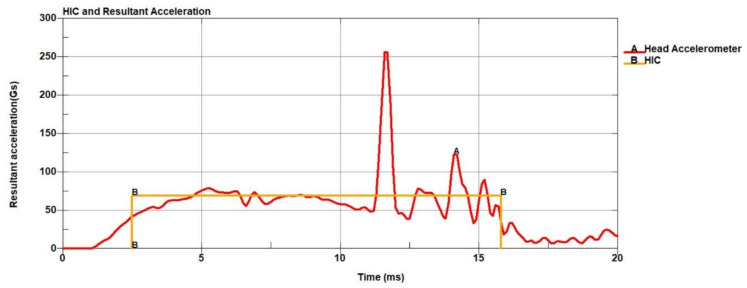
HIC and resultant acceleration (g) in time (ms) plot (HIC = 536).

**Figure 9:**
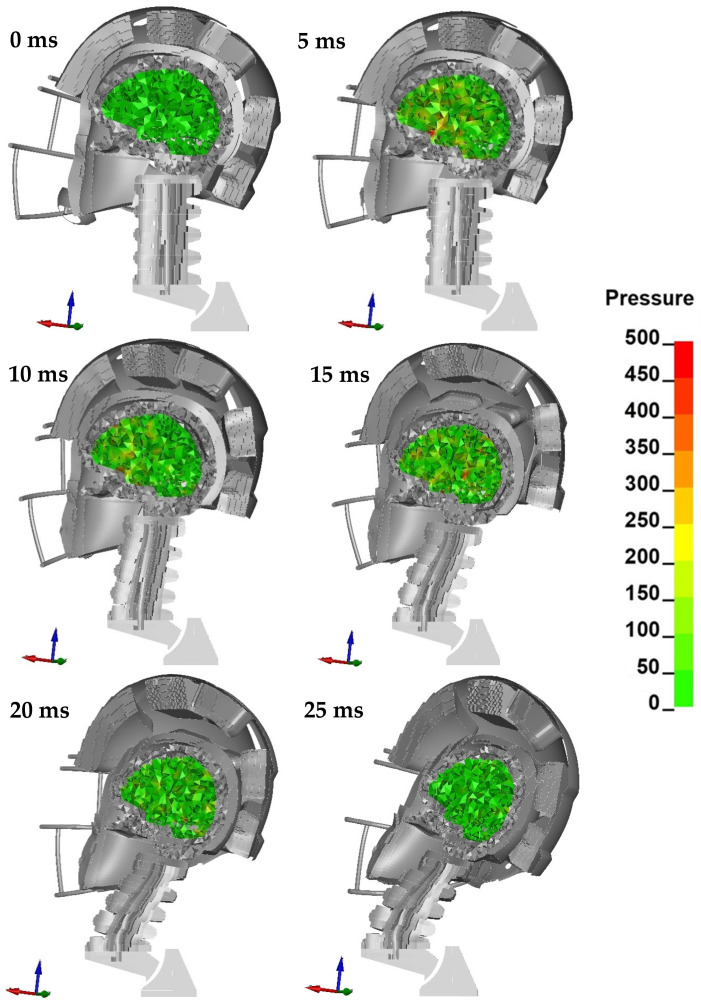
Head kinematics during the numerical test, configuration A, with the cross-section in the sagittal plane and showing the hydrostatic pressure (kPa) in the brain.

The course of simulation in Figure 7 presents the general kinematics of the helmet after being hit with the impactor. Figure 8 depicts the resultant linear accelerations in time and presents the HIC value graphically. Figure 9 presents a cross-section in the sagittal plane during the simulation. Hydrostatic pressure (kPa) in the brain is highlighted. The remaining configurations (AP–UT) are presented in Appendix A.

## 4. Discussion

The market for numerical models is expanding at an excessive pace. Nevertheless, there are few models verified by experimental studies and that include the entire Hybrid III Head-Neck Model. Many numerical models are duplicated and are based on the same mechanical properties for individual tissue components. A similar situation applies to tests for helmets, where often there is no verification. The subject of head protection is a critical topic in terms of preventing brain trauma. Acquired football helmets may not fully protect the player, as this study showed. Other authors have made similar conclusions [19,20,22,23,24,29,30,31,37,38]. Since traumatic brain injury is associated with a large number of neuropathological changes and mental impairment, it is the subject of research by many authors. Neurological changes are a diagnostic challenge and are not fully understood. Hence, there are many divergent research results in the literature.

It is known that physical damage in the structure of the brain itself, as well as the subsequent disturbances in biochemical pathways, can cause many mental and psychosomatic disorders. The use of numerical brain models and their analysis of the response to the load gives insight into the biomechanical tissue response. In this study, based on mechanical values in brain tissues, a football helmet’s safety level under various impact configurations was determined. The authors analyzed the biomechanical response to the impact in the form of the head injury criterion and hydrostatic pressure. It should be noted here that HIC has recently been widely criticized in the literature because it is based on linear accelerations and does not give any insight into the angular response of tissues. These suggestions were confirmed by this paper. We also proved that ICP and HIC values do not correlate. This implies that more complex calculated and validated numerical results should be translated into the acceleration-based injury criteria. Hence, many researchers are looking for appropriate measures for evaluating biomechanical tissues. In this context, numerical models using finite elements, supported by experimental research, play a crucial role. We emphasize the crucial role of hydrostatic pressure as an important parameter in the analysis of brain injuries. The brain is enclosed in a relatively rigid skull capsule, and repeatable shaking may cause pressure on other tissues, which translates into a change in intracranial pressure. Hence, subsequent research works should, inter alia, base the results of their numerical tests on pressure changes, at the same time incorporating experimental tests, which will constitute the basis for the verification of the simulation. Overall, the results underline that it is necessary to continue the investigation on related brain injury mechanisms in helmet tests in order to minimalize the probability of injury. The use of validated discrete head models seems to be one of the best ways to evaluate the behavior of the brain during collisions. The finite element method is now frequently used to simulate or recreate accidents. Moreover, this tool enables the verification of various constructions, such as passive safety systems in vehicles or helmets. Developing more accurate models representing the human body allows one to simulate the injuries that may occur. With this technology, it is possible to design and develop protective gear with maximum efficiency. It is possible that in the near future, the numerical analysis will correctly mimic the validation tests of protective gear and constructions. This is an excellent way to study body injuries under different loads and injury mechanisms. Once an actual brain model is developed, it will be possible to study the brain’s reaction to different kinematics and loading inputs, analyzing the brain response in terms of parameters such as intracranial pressure and brain strain.

## 5. Conclusions

In this study, a new approach to helmet simulation is presented. Inspecting intracranial pressure is a very beneficial methodology in brain analysis. The information gathered in the research can strongly influence the design process. This research should be continued and broadened with more standardized tests. HIC, which originated in automotive dummy tests in the field of motor vehicle regulation, is currently commonly used in various head injury studies, including TBI and concussion. This paper confirms that HIC is used regardless of the specific type of head impact. The understanding of brain behavior during the impact will allow implementation of new energy-absorbing materials and possible concussion diagnosis. Another suggestion is to replace the NOCSAE headform and HIII head model with new dummies. A good example is the THUMS dummy, as its head was developed with a simplified brain structure.

This research raises concern about applying the HIC to predict brain injury in direct head impacts in American football. This study proved that further research definitely should be conducted on helmets. Certification tests for motorbike, race car, lacrosse, American football and hockey helmets are all different, but they share a common assessment. Drop impacts, and pneumatic ram impacts are based on linear and rotational accelerations. The NOCSAE organization validates sports equipment as well as industrial safety, fire and emergency equipment. Firefighters’ helmets may also be analyzed for their protection of brain tissues. Based on this research, the displacement of the brain lobes, principal strain and stress should be investigated. Another interesting point would be an analysis of forces acting on the neck and their interpretation in terms of neck injury criteria (NIC) or normalized neck injury criteria (Nij). With that being researched, it will be possible to design new energy-absorbing technology that will help to minimize the probability of head injury.

## Figures and Tables

**Table 1:** Mechanical properties for each component of the head (presented in details in [66]).

Element	Young’s (E) or Bulk Modulus (K) [MPa]	Density [kg/m^3^]	Poisson’s Ratio
Skull	E = 15,000.0	2000	0.22
Dura mater	E = 31.5	1130	0.45
Cerebrospinal fluid	K = 2200.0	1000	0.49
Superior sagittal sinus	E = 28.2	1040	0.45
Falx cerebri and cerebellar tentorium	E = 31.5	1130	0.45
Brain tissue	K = 1130.0	1040	not applicable

**Table 2:** Mechanical properties of bridging veins (samples from people older than 50 years of age [67]).

Bridging Veins Region	Young’s Modulus [MPa]	Density [kg/m^3^]	Poisson’s Ratio
Frontal	56.45	1130	0.45
Parietal	94.09	1130	0.45
Occipital	97.21	1130	0.45

**Table 3:** Material properties of the helmet and impactor components [68].

Part	$\mathrm{Density}\;\left[\frac{kg}{m^{3}}\right]$	Young’s Modulus [MPa]	Poisson’s Ratio	Material Model in LS-DYNA
**Helmet**				
FACEMASK	8546.0	210,000	0.3	ELASTIC
SHELL	1095.0	1565	0.3	ELASTIC
PADDING (FRONT)	170.5	3	-	FU_CHANG_FOAM_LOG_INTERPOLATION
PADDING (TOP, SIDES)	70.0 95.0	20	-	FU_CHANG_FOAM_LOG_INTERPOLATION
PADDING (BACK)	100.0	200	-	FU_CHANG_FOAM_LOG_INTERPOLATION
**Impactor**				
NYLON END CAP	1140.0	2410	0.4	ELASTIC
VINYL NITRILE	122.6	1000	-	FU_CHANG_FOAM_LOG_INTERPOLATION
BACKING PLATE	6899.0	200,000	0.3	RIGID
RAM	140,700	200,000	0.3	RIGID

**Table 4:** Investigated approach setup.

Configuration	Simulation	Configu-Ration	Simulation
A	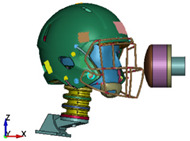	D	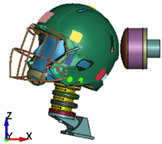
AP	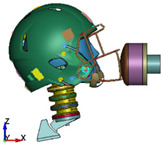	F	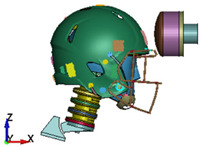
B	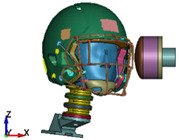	R	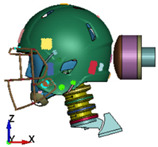
C	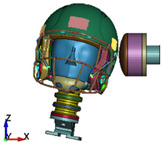	UT	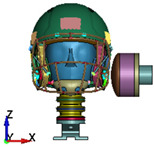

**Table 5:** Comparison of the HIC score for the Hybrid III dummy to the maximal value of hydrostatic pressure for αHEAD.

Configuration	HIC Score (HIII Model)	Hydrostatic Pressure [kPa] at 6 ms after Impact [ms] (αHEAD Model)		Ratio of Finite Elements Exceeding Threshold Criterion (237 kPa)
A	536	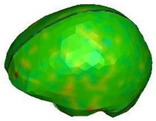	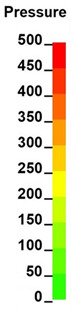	29.18%
P	650	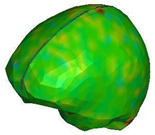		29.62%
B	449	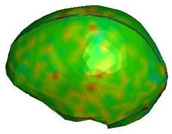		24.20%
C	557	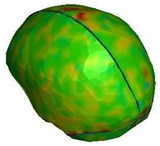		21.60%
D	594	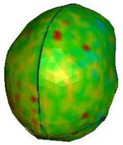	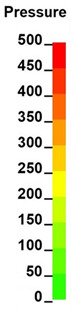	20.64%
F	403	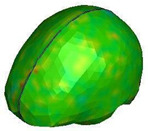		25.55%
R	731	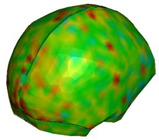		25.42%
UT	304	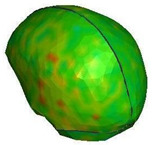		25.66%

**Figure A1:**
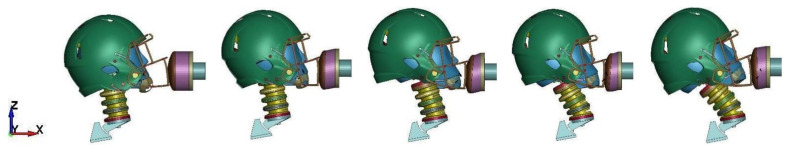
The course of simulation (0–20 ms, 5 ms interval) in configuration AP.

**Figure A2:**
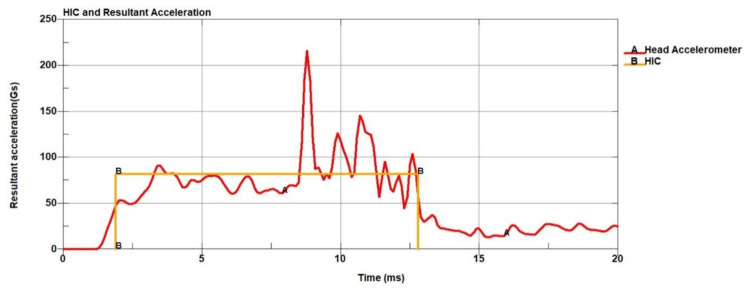
HIC and Resultant Acceleration (g) in time (ms) plot (HIC = 650).

**Figure A3:**
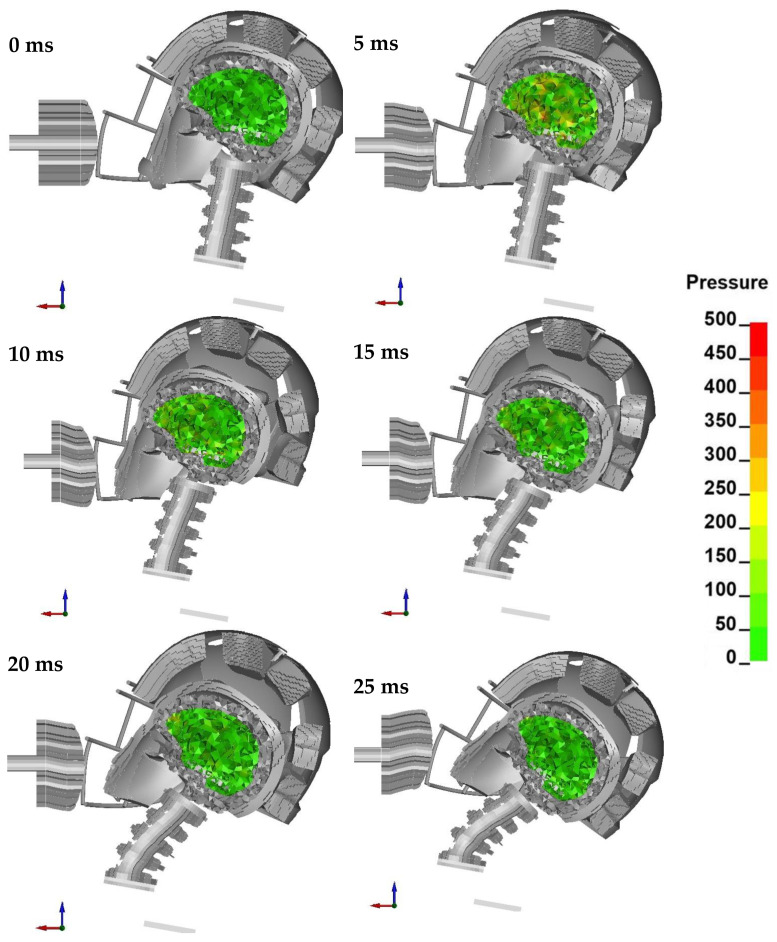
Head kinematics during the numerical test, configuration AP, with the cross-section in the sagittal plane and showing the hydrostatic pressure (kPa) in the brain.

**Figure A4:**
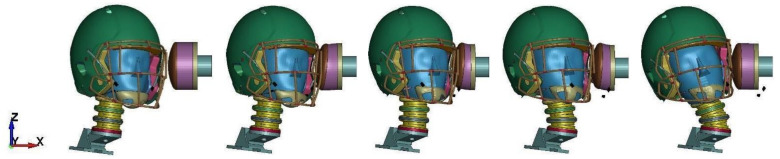
Course of simulation (0–20 ms, 5 ms interval) in configuration B.

**Figure A5:**
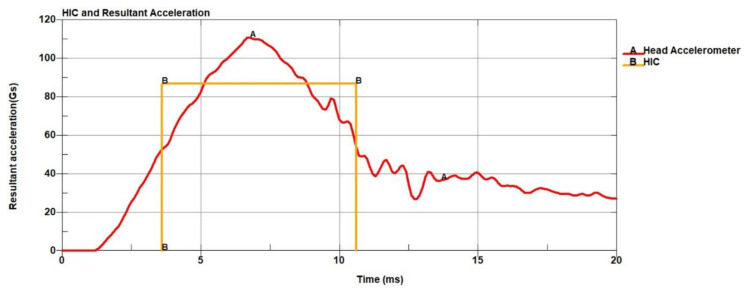
HIC and resultant acceleration (g) in time (ms) plot (HIC = 499).

**Figure A6:**
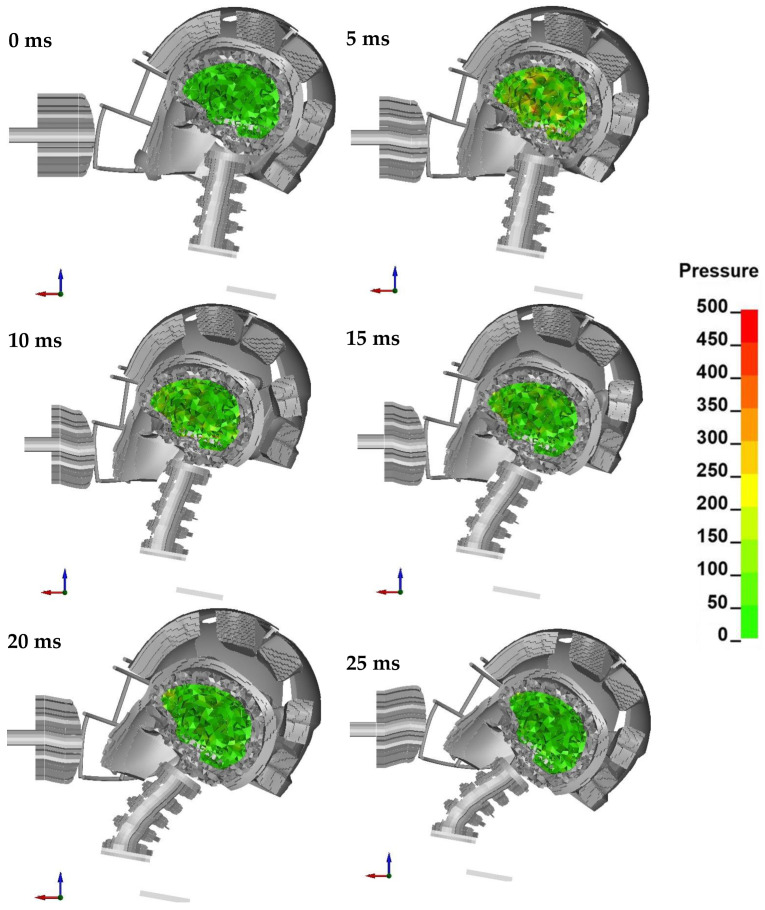
Head kinematics during the numerical test, configuration B, with the cross-section in the median plane and showing the hydrostatic pressure (kPa) in the brain.

**Figure A7:**
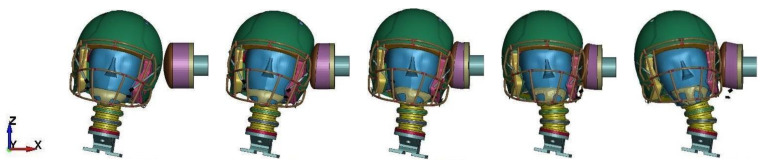
Course of simulation (0–20 ms, 5 ms interval) in configuration C.

**Figure A8:**
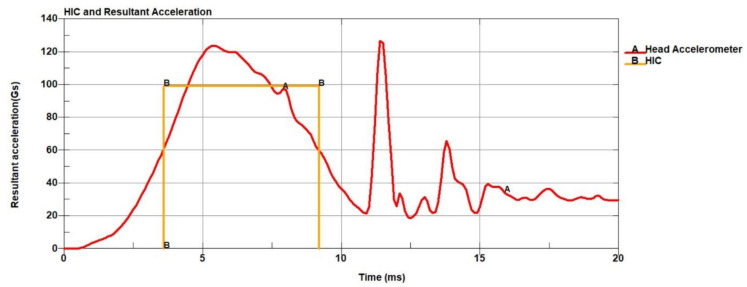
HIC and resultant acceleration (g) in time (ms) plot (HIC = 557).

**Figure A9:**
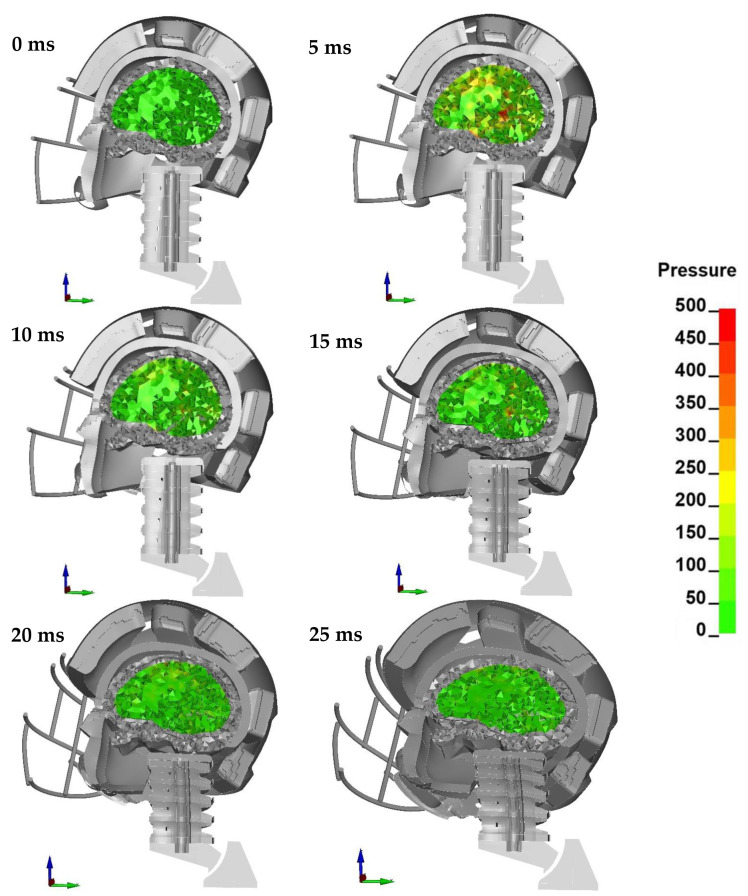
Head kinematics during the numerical test, configuration C, with the cross-section in the median plane and showing the hydrostatic pressure (kPa) in the cerebrum.

**Figure A10:**
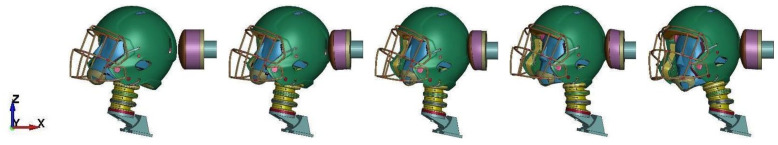
Course of simulation (0-20 ms, 5 ms interval) in configuration D.

**Figure A11:**
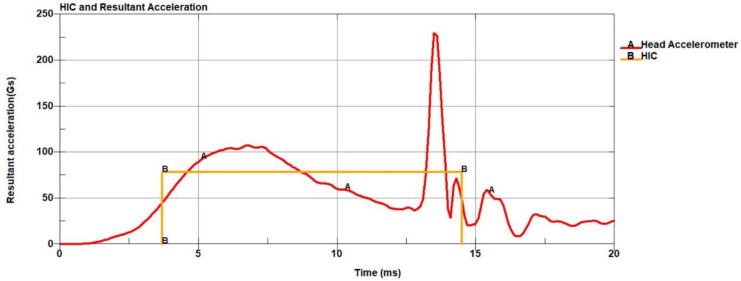
HIC and resultant acceleration (g) in time (ms) plot (HIC = 594).

**Figure A12:**
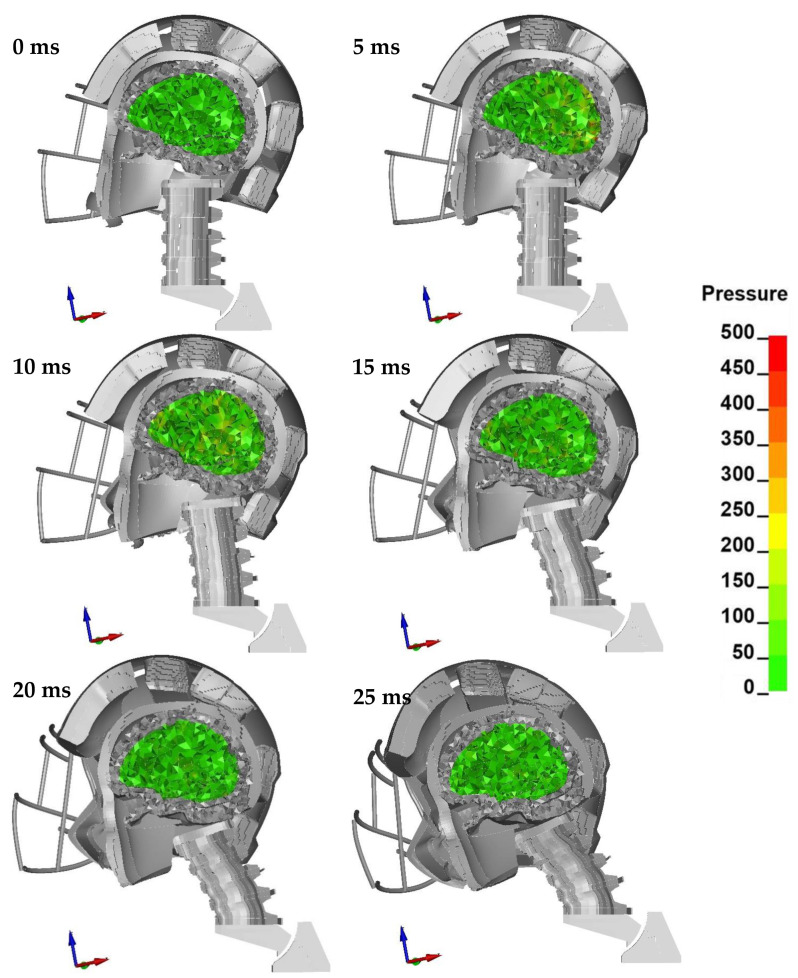
Head kinematics during the numerical test, configuration D, with the cross-section in the median plane and showing the hydrostatic pressure (kPa) in the cerebrum.

**Figure A13:**
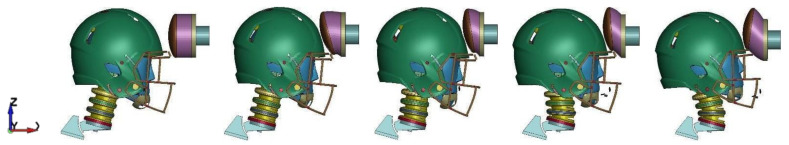
The course of simulation (0–20 ms, 5 ms interval) in configuration F.

**Figure A14:**
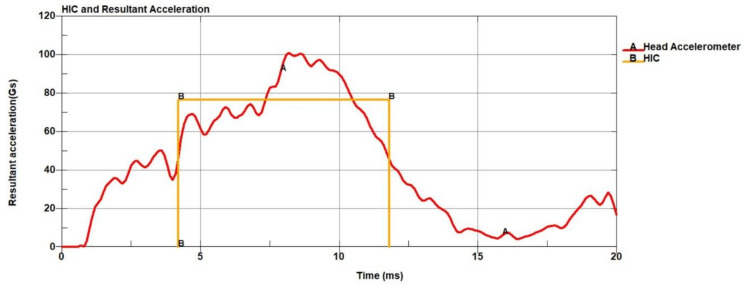
HIC and resultant acceleration (g) in time (ms) plot (HIC = 403).

**Figure A15:**
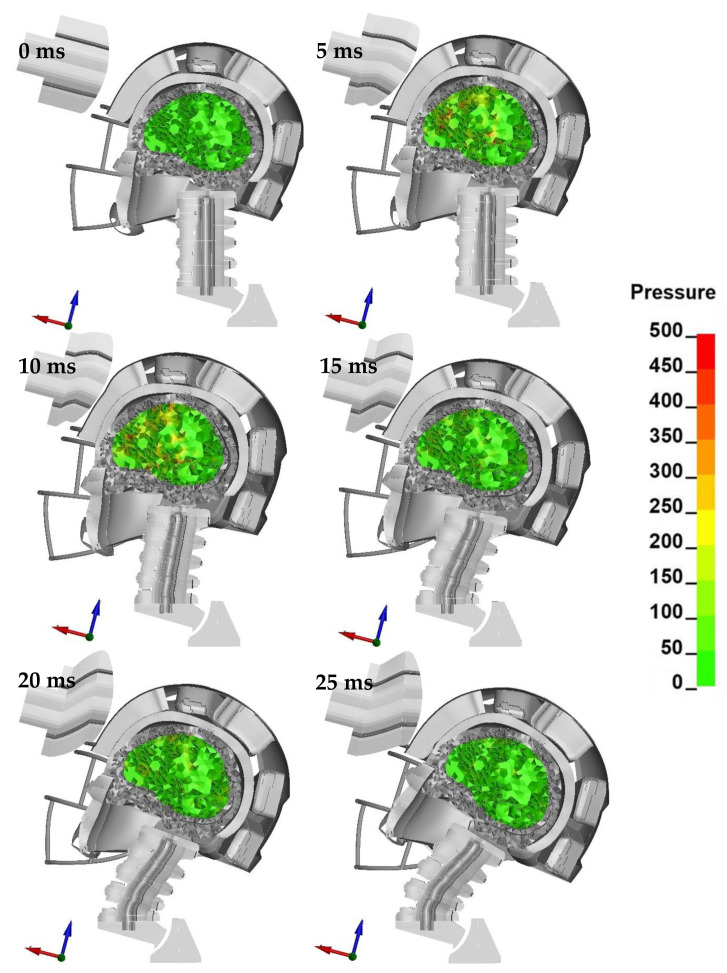
Head kinematics during the numerical test, configuration F, with the cross-section in the median plane and showing the hydrostatic pressure (kPa) in the cerebrum.

**Figure A16:**
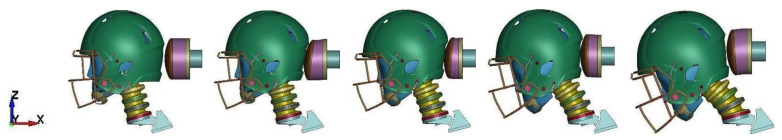
The course of simulation (0–20 ms, 5 ms interval) in configuration R.

**Figure A17:**
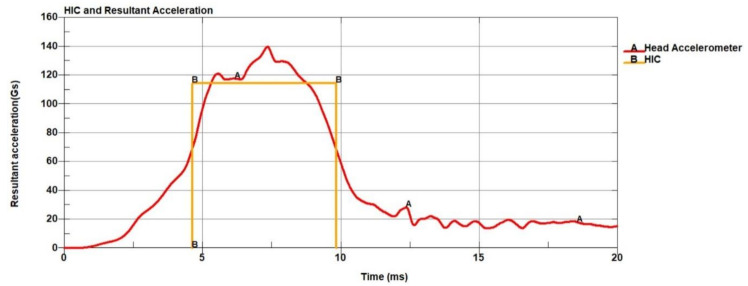
HIC and resultant acceleration (g) in time (ms) plot (HIC = 731).

**Figure A18:**
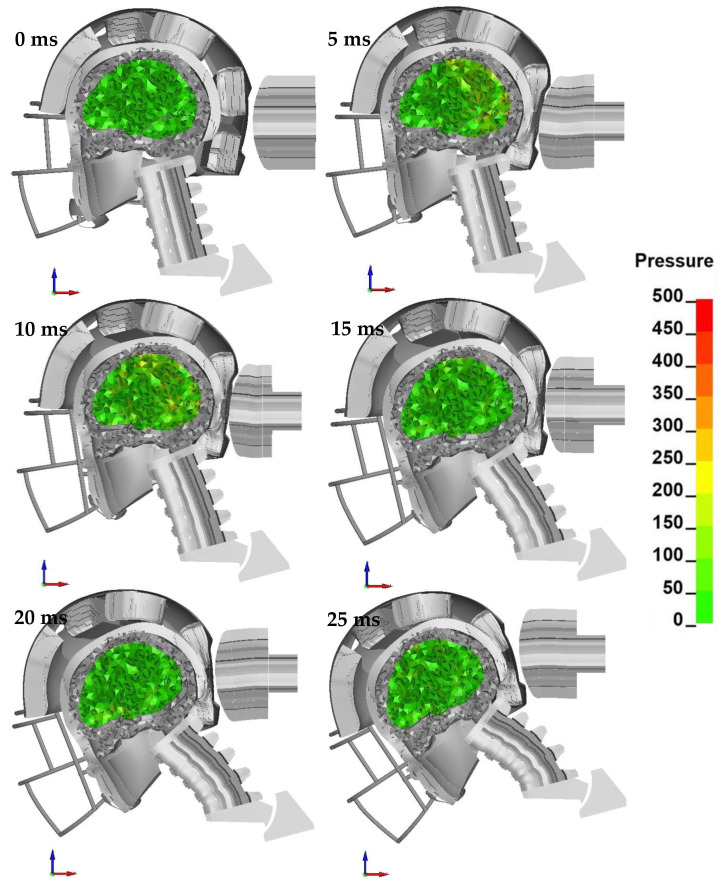
Head kinematics during the numerical test, configuration R, with the cross-section in the median plane and showing the hydrostatic pressure (kPa) in the cerebrum.

**Figure A19:**
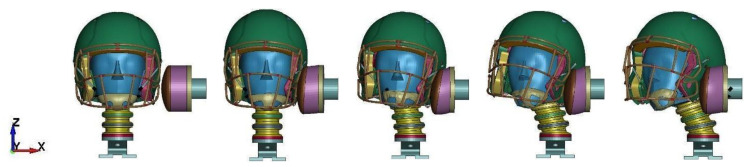
The course of simulation (0–20 ms, 5 ms interval) in configuration UT.

**Figure A20:**
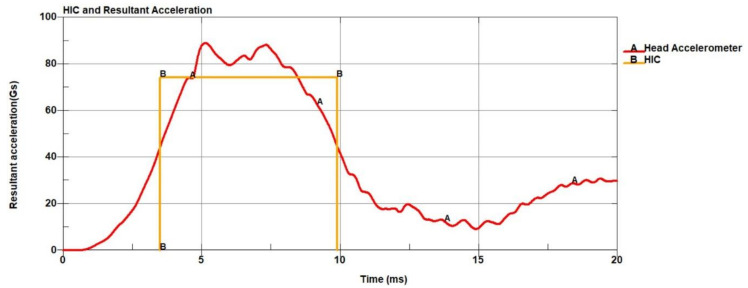
HIC and resultant acceleration (g) in time (ms) plot (HIC = 304).

**Figure A21:**
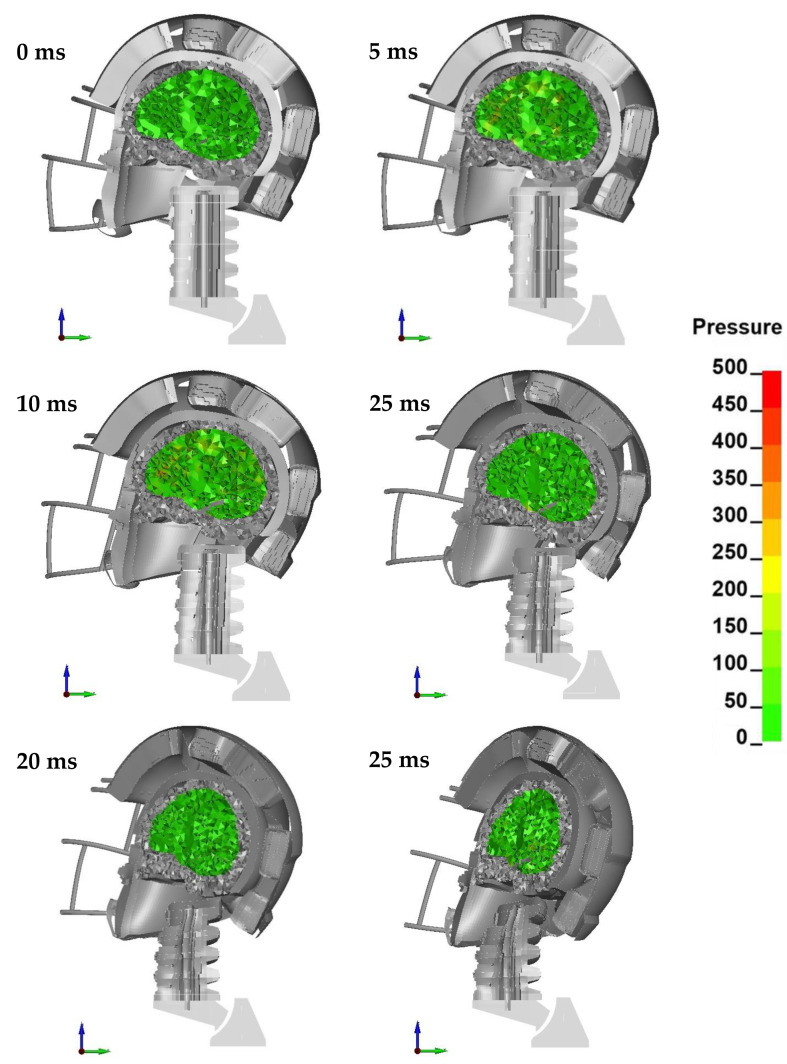
Head kinematics during the numerical test, configuration UT, with the cross-section in the median plane and showing the hydrostatic pressure (kPa) in the cerebrum.

## Data Availability

Not applicable.

## References

[1] Saal J.A. (1991). Common American Football Injuries. Sport. Med..

[2] Kucera K.L., Yau R.K., Register-Mihalik J., Marshall S.W., Thomas L.C., Wolf S., Cantu R.C., Mueller F.O., Guskiewicz K.M. (2017). Traumatic Brain and Spinal Cord Fatalities Among High School and College Football Players—United States, 2005–2014. MMWR. Morb. Mortal. Wkly. Rep..

[3] Casson I.R., Viano D.C., Powell J.W., Pellman E.J. (2010). Twelve Years of National Football League Concussion Data. Sport. Health A Multidiscip. Approach.

[4] Boden B.P., Tacchetti R.L., Cantu R.C., Knowles S.B., Mueller F.O. (2007). Catastrophic head injuries in high school and college football players. Am. J. Sports Med..

[5] Gessel L.M., Fields S.K., Collins C.L., Dick R.W., Comstock R.D. (2007). Concussions among United States high school and collegiate athletes. J. Athl. Train..

[6] Liu Y., Domel A.G., Yousefsani S.A., Kondic J., Grant G., Zeineh M., Camarillo D.B. (2020). Validation and Comparison of Instrumented Mouthguards for Measuring Head Kinematics and Assessing Brain Deformation in Football Impacts. Ann. Biomed. Eng..

[7] Pellman E.J., Viano D.C. (2006). Concussion in professional football. Neurosurg. Focus.

[8] Arnason A., Sigurdsson S.B., Gudmundsson A., Holme I., Engebretsen L., Bahr R. (2004). Risk Factors for Injuries in Football. Am. J. Sports Med..

[9] McIntosh A.S., McCrory P. (2005). Preventing head and neck injury. Br. J. Sports Med..

[10] Fernandes F.A.O., Alves de Sousa R.J., Ptak M., Wilhelm J. (2020). Certified Motorcycle Helmets: Computational Evaluation of the Efficacy of Standard Requirements with Finite Element Models. Math. Comput. Appl..

[11] Jamroziak K., Bajkowski M., Bocian M., Polak S., Magier M., Kosobudzki M., Stepien R. (2019). Ballistic Head Protection in the Light of Injury Criteria in the Case of the Wz.93 Combat Helmet. Appl. Sci..

[12] Wilhelm J., Ptak M., Rusiński E. (2017). Simulated depiction of head and brain injuries in the context of cellularbased materials in passive safety devices. Sci. Journals Marit. Univ. Szczecin.

[13] Varela M.M., Fernandes F.A.O., Alves de Sousa R.J. (2020). Development of an Eco-Friendly Head Impact Protection Device. Appl. Sci..

[14] Kraft R.H., Mckee P.J., Dagro A.M., Grafton S.T. (2012). Combining the Finite Element Method with Structural Connectome-based Analysis for Modeling Neurotrauma: Connectome Neurotrauma Mechanics. PLoS Comput. Biol..

[15] Aare M., Kleiven S., Halldin P. (2004). Injury criteria for oblique helmet impacts. Int. J. Crashworthiness.

[16] Prasad P., Mertz H.J. (1985). The position of the United States delegation to the ISO Working Group 6 on the use of HIC in the automotive environment. SAE Trans..

[17] Marjoux D., Baumgartner D., Deck C., Willinger R. (2008). Head injury prediction capability of the HIC, HIP, SIMon and ULP criteria. Accid. Anal. Prev..

[18] Takhounts E.G., Craig M.J., Moorhouse K., McFadden J., Hasija V. (2013). Development of brain injury criteria (BrIC). Stapp Car Crash J..

[19] Post A., Kendall M., Cournoyer J., Karton C., Oeur R.A., Dawson L., Hoshizaki T.B. (2018). Brain tissue analysis of impacts to American football helmets. Comput. Methods Biomech. Biomed. Engin..

[20] Darling T., Muthuswamy J., Rajan S.D. (2016). Finite element modeling of human brain response to football helmet impacts. Comput. Methods Biomech. Biomed. Engin..

[21] Barker J., Corrales M., Gierczycka D., Bruneau D., Bustamante M.C., Cronin D. Contribution of Energy-Absorbing Structures to Head Kinematics in Football Helmet Impacts. Proceedings of the International Research Conference on the Biomechanics of Impacts (IRCOBI).

[22] Corrales M.A., Gierczycka D., Barker J., Bruneau D., Bustamante M.C., Cronin D.S. (2020). Validation of a Football Helmet Finite Element Model and Quantification of Impact Energy Distribution. Ann. Biomed. Eng..

[23] Lamb L., Hoshizaki T.B. (2009). Deformation mechanisms and impact attenuation characteristics of thin-walled collapsible air chambers used in head protection. Proc. Inst. Mech. Eng. Part H J. Eng. Med..

[24] Dean S.W., Hoshizaki T.B., Post A. (2009). Impact Attenuation Characteristics of Thin Walled Collapsible Air Chambers for Use in Protective Helmets. J. ASTM Int..

[25] Schwarz A. Helmet Design Absorbs Shock in a New Way. https://www.nytimes.com/2007/10/27/sports/football/27helmets.html.

[26] Sybilski K., Małachowski J. (2019). Sensitivity study on seat belt system key factors in terms of disabled driver behavior during frontal crash. Acta Bioeng. Biomech..

[27] Hazay M., Bakos B., Toth P.J., Buki A., Bojtar I. (2018). Optimization of decompressive craniectomy based on finite element simulations. Acta Polytech. CTU Proc..

[28] Cobb B.R., Zadnik A.M., Rowson S. (2016). Comparative analysis of helmeted impact response of Hybrid III and National Operating Committee on Standards for Athletic Equipment headforms. Proc. Inst. Mech. Eng. Part P J. Sport. Eng. Technol..

[29] Viano D.C., Withnall C., Halstead D. (2012). Impact Performance of Modern Football Helmets. Ann. Biomed. Eng..

[30] Johnston J.M., Ning H., Kim J.-E., Kim Y.-H., Soni B., Reynolds R., Cooper L., Andrews J.B., Vaidya U. (2015). Simulation, fabrication and impact testing of a novel football helmet padding system that decreases rotational acceleration. Sport. Eng..

[31] Dymek M., Peliński J. (2020). Numerical simulation of professional American football helmet with head model. Aktual. Probl. Biomech..

[32] King A.I., Yang K.H., Zhang L., Hardy W. Is head injury caused by linear or angular acceleration?. Proceedings of the International Research Conference on the Biomechanics of Impacts (IRCOBI).

[33] Wu L.C., Kuo C., Loza J., Kurt M., Laksari K., Yanez L.Z., Senif D., Anderson S.C., Miller L.E., Urban J.E. (2018). Detection of American Football Head Impacts Using Biomechanical Features and Support Vector Machine Classification. Sci. Rep..

[34] Hernandez F., Giordano C., Goubran M., Parivash S., Grant G., Zeineh M., Camarillo D. (2019). Lateral impacts correlate with falx cerebri displacement and corpus callosum trauma in sports-related concussions. Biomech. Model. Mechanobiol..

[35] Tierney G.J., Kuo C., Wu L., Weaving D., Camarillo D. (2020). Analysis of head acceleration events in collegiate-level American football: A combination of qualitative video analysis and in-vivo head kinematic measurement. J. Biomech..

[36] Bailey A.M., Sanchez E.J., Park G., Gabler L.F., Funk J.R., Crandall J.R., Wonnacott M., Withnall C., Myers B.S., Arbogast K.B. (2020). Development and Evaluation of a Test Method for Assessing the Performance of American Football Helmets. Ann. Biomed. Eng..

[37] Bailey A.M., McMurry T.L., Cormier J.M., Funk J.R., Crandall J.R., Mack C.D., Myers B.S., Arbogast K.B. (2020). Comparison of Laboratory and On-Field Performance of American Football Helmets. Ann. Biomed. Eng..

[38] Giudice J.S., Caudillo A., Mukherjee S., Kong K., Park G., Kent R., Panzer M.B. (2020). Finite Element Model of a Deformable American Football Helmet Under Impact. Ann. Biomed. Eng..

[39] Tierney G.J., Simms C. (2019). Predictive Capacity of the MADYMO Multibody Human Body Model Applied to Head Kinematics During Rugby Union Tackles. Appl. Sci..

[40] Hasegawa Y., Kawasaki T., Miyazaki Y., Sobue S., Kaketa T., Gonda Y., Kaneko K. (2020). Changes of the Cervical Spine in Response to Head-first Impact in Rugby: A Finite Element Analysis. Juntendo Med. J..

[41] Cogoluenhes L., Evin M., Forodighasemabadi A., Wei W., Thollon L., Llari M. (2019). A modelisation of quantification of head and neck risks associated with tackles in rugby union. Comput. Methods Biomech. Biomed. Engin..

[42] O’Keeffe E., Kelly E., Liu Y., Giordano C., Wallace E., Hynes M., Tiernan S., Meagher A., Greene C., Hughes S. (2020). Dynamic Blood–Brain Barrier Regulation in Mild Traumatic Brain Injury. J. Neurotrauma.

[43] Giudice J.S., Park G., Kong K., Bailey A., Kent R., Panzer M.B. (2019). Development of Open-Source Dummy and Impactor Models for the Assessment of American Football Helmet Finite Element Models. Ann. Biomed. Eng..

[44] Bustamante M.C., Bruneau D., Barker J.B., Gierczycka D., Coralles M.A., Cronin D.S. (2019). Component-Level Finite Element Model and Validation for a Modern American Football Helmet. J. Dyn. Behav. Mater..

[45] Ptak M. (2019). Method to Assess and Enhance Vulnerable Road User Safety during Impact Loading. Appl. Sci..

[46] Schoell S.L., Weaver A.A., Urban J.E., Jones D.A., Stitzel J.D., Hwang E., Reed M.P., Rupp J.D., Hu J. (2015). Development and Validation of an Older Occupant Finite Element Model of a Mid-Sized Male for Investigation of Age-related Injury Risk. Stapp Car Crash J..

[47] Greenberg M. (2019). Greenberg Handbook of Neurosurgery.

[48] Mondello S., Guedes V.A., Lai C., Czeiter E., Amrein K., Kobeissy F., Mechref Y., Jeromin A., Mithani S., Martin C. (2020). Circulating Brain Injury Exosomal Proteins following Moderate-to-Severe Traumatic Brain Injury: Temporal Profile, Outcome Prediction and Therapy Implications. Cells.

[49] Champagne A.A., Coverdale N.S., Fernandez-Ruiz J., Mark C.I., Cook D.J. (2020). Compromised resting cerebral metabolism after sport-related concussion: A calibrated MRI study. Brain Imaging Behav..

[50] Russman A., Figler R., Oh S.-H., Lowe M., Jones S. (2015). 7 Tesla Brain MRI Characteristics Among Concussion Patients (P7.162). Neurology.

[51] Ken Belson New York Times Aaron Hernandez Had Severe C.T.E. When He Died at Age 27. https://www.nytimes.com/2017/09/21/sports/aaron-hernandez-cte-brain.html.

[52] Boston University Research: CTE Center Frequently Asked Questions about CTE. https://www.bu.edu/cte/about/frequently-asked-questions/.

[53] Mez J., Daneshvar D.H., Kiernan P.T., Abdolmohammadi B., Alvarez V.E., Huber B.R., Alosco M.L., Solomon T.M., Nowinski C.J., McHale L. (2017). Clinicopathological Evaluation of Chronic Traumatic Encephalopathy in Players of American Football. JAMA.

[54] Fernandes F.A.O., Sousa R.J.A.D. (2015). Head injury predictors in sports trauma—A state-of-the-art review. Proc. Inst. Mech. Eng. Part H J. Eng. Med..

[55] Ji S., Zhao W., Ford J.C., Beckwith J.G., Bolander R.P., Greenwald R.M., Flashman L.A., Paulsen K.D., McAllister T.W. (2015). Group-Wise Evaluation and Comparison of White Matter Fiber Strain and Maximum Principal Strain in Sports-Related Concussion. J. Neurotrauma.

[56] Ghazi K., Wu S., Zhao W., Ji S. (2020). Instantaneous Whole-brain Strain Estimation in Dynamic Head Impact. J. Neurotrauma.

[57] Echemendia R.J., Meeuwisse W., McCrory P., Davis G.A., Putukian M., Leddy J., Makdissi M., Sullivan S.J., Broglio S.P., Raftery M. (2017). The Sport Concussion Assessment Tool 5th Edition (SCAT5). Br. J. Sports Med..

[58] Saunders R.L. (1984). The Second Impact in Catastrophic Contact-Sports Head Trauma. JAMA J. Am. Med. Assoc..

[59] Jordan B.D., Jahre C., Hauser W.A., Zimmerman R.D., Zarrelli M., Lipsitz E.C., Johnson V., Warren R.F., Tsairis P., Folk F.S. (1992). CT of 338 active professional boxers. Radiology.

[60] Ratajczak M., Ptak M., Chybowski L., Gawdzińska K., Będziński R. (2019). Material and Structural Modeling Aspects of Brain Tissue Deformation under Dynamic Loads. Materials.

[61] Costa J.M.C., Fernandes F.A.O., Alves de Sousa R.J. (2020). Prediction of subdural haematoma based on a detailed numerical model of the cerebral bridging veins. J. Mech. Behav. Biomed. Mater..

[62] Fernandes F.A.O., Tchepel D., Alves de Sousa R.J., Ptak M. (2018). Development and validation of a new finite element human head model: Yet another head model (YEAHM). Eng. Comput..

[63] Wilhelm J., Ptak M., Fernandes F.A.O., Kubicki K., Kwiatkowski A., Ratajczak M., Sawicki M., Szarek D. (2020). Injury Biomechanics of a Child’s Head: Problems, Challenges and Possibilities with a New aHEAD Finite Element Model. Appl. Sci..

[64] Giudice J., Sebastian K., Kong A., Caudillo S., Mukherjee M.B.P. Finite Element Models of Helmet Assessment Tools 2018. https://biocorellc.com/finite-element-models/.

[65] Kleiven S., Von Holst H. (2002). Consequences of head size following trauma to the human head. J. Biomech..

[66] Kleiven S. (2002). Finite Element Modeling of the Human Head. Ph.D. Thesis.

[67] Horanin-Dusza M. (2009). The Analysis of the Biomechanical and Histological Properties of Cerebral Bridging Veins in Alcoholics and Nonalcoholics—The Importance in the Subdural Hematomas Etiology (in Polish). Ph.D. Thesis.

[68] M. Fahlstedt M., Arnesen E., Jungstedt P.H. Finite Element Model of 2016 Riddell Speed Classic (Safety Equipment Institute model R41179) 2018. https://biocorellc.com/finite-element-models/.

[69] (2019). Standard Performance Specification for Newly Manufactured Football Helmets.

[70] Gwin J.T., Diamond S.G., Halstead P.D., Greenwald R.M. (2010). An Investigation of the NOCSAE Linear Impactor Test Method Based on In Vivo Measures of Head Impact Acceleration in American football. J. Biomech. Eng..

[71] Zhang L., Yang K.H., King A.I. (2001). Comparison of brain responses between frontal and lateral impacts by finite element modeling. J. Neurotrauma.

